# Changes in the Lodging-Related Traits along with Rice Genetic Improvement in China

**DOI:** 10.1371/journal.pone.0160104

**Published:** 2016-07-28

**Authors:** Guanglong Zhu, Guohui Li, Depeng Wang, Shen Yuan, Fei Wang

**Affiliations:** National Key Laboratory of Crop Genetic Improvement, MOA Key Laboratory of Crop Ecophysiology and Farming System in the Middle Reaches of the Yangtze River, College of Plant Science and Technology, Huazhong Agricultural University, Wuhan, Hubei, 430070, China; New Mexico State University, UNITED STATES

## Abstract

Rice yield potential was greatly improved since the green revolution, but the occurrence of lodging often restricts the achievement of potential yield. Currently, it is still obscure about how the lodging-related traits change along with the genetic improvement in yield potential of rice, although much efforts have been devoted to study the trend of and physiological mechanisms underlying changes in grain yield. Therefore, fourteen rice mega-varieties that were released and disseminated from 1930s to 2005 in China were investigated through a two-year experiment in the field condition. The results showed that large genotypic differences in lodging-related morphological traits were observed among these varieties. Lodging index (LI) of semi-dwarf varieties was significantly lower compared with that of SLX(Shenglixian). There were significant differences in LI among the semi-dwarf varieties, but no relationship between LI and the release year was found. Bending moment (BM) of semi-dwarf varieties released in 1940s-1980s was significantly lower than that of SLX. However, varieties released after 1980s had similar bending moment with SLX, but significantly higher breaking resistance (BR). The increase in both BM and BR after 1980s was related with the increase in internode diameter (ND) and stem fresh weight. Overall, this study disclosed the changing pattern of lodging-related traits in the genetic improvement of rice, and suggested that further increase in ND, internode dry weight (NDW) and dry weight per unit length (DWUL) of lower internode in modern super rice variety could effectively enhance lodging resistance and bring down LI.

## Introduction

Rice (*Oryza sativa* L.) is a major staple food for more than half of world's population [1]. Yield potential of rice was greatly improved since the green revolution due to the development of semi-dwarf varieties in the late 1950s, three-line hybrids in the late 1970s, and super-hybrid rice since 1996 [2–3]. The "three-line system" for hybrid rice production includes cytoplasmic male-sterile line, maintainer line and restorer line [2]. The success of super-hybrid rice was due to a combination of superior agronomic characteristics of IRRI’s New Plant Type and inter-subspecific heterosis [4], which led to a 12% advantage in yield potential compared with ordinary hybrids and inbred varieties [5]. The higher grain yields of super-hybrid rice are attributed to improvement in both source and sink capacity [6], and improvement in crop management [4].

Lodging is one of the factors causing grain yield reduction for most cereal crops, and could reduce grain yield by up to 50%, especially for rice in high-yielding environments [7]. Occurrence of lodging is common after strong wind accompanied by heavy rains during the grain filling period. Recently, over-luxuriant growth due to excessive nitrogen application, high planting density, and global warming have led to increased lodging occurrence [8]. In a lodged plant community, the normal canopy structure is destroyed, leading to reduced photosynthetic ability and dry matter production [9]. Severe lodging prevents the transport of water, nutrients, and assimilates through the xylem and phloem, resulting in a reduction in assimilates for grain filling [10]. Furthermore, high moisture levels in a lodged plant community may be favorable for fungal growth and the occurrence of diseases, which have detrimental effects on grain quality [11]. In situ germination may occur in lodged plants due to conducive environment especially for cultivars with weak seed dormancy. As a result, lodging could cause great losses in both grain yield and quality. In addition, it also causes difficulties in harvest operations, increases demand for grain drying, and consequently results in increased production cost [12].

During the green revolution, dwarfing genes were introduced into wheat and rice varieties to reduce height in order to improve lodging resistance and yield potential of cereal crops [13–14]. Recent studies, however, suggest that reduced plant height of semi-dwarf rice and wheat may limit canopy photosynthesis and biomass production, and therefore, resulted in grain yield stagnation [15–16]. Miralles and Slafer (1995) showed that dwarf wheat may reduce radiation use efficiency in high radiation condition due to more prostrate leaves[17]. Under dry conditions shorter plants may accumulate fewer water soluble carbohydrates in the stem which may reduce the crop’s buffering capacity against poor grain filling conditions. Finally dwarf plants may be more susceptible to certain diseases and weeds because the spread of splash-borne foliar disease is more rapid in short plants [18] and shorter plants have a weak capacity to control weeds [19]. Therefore, there is a contradictory relationship between grain yield potential and lodging resistance [20], and it has been shown that a reduction of approximately 6 cm in plant height could offset an increase of 1 t ha^-1^ in grain yield potential [21]. If height decreases have not kept pace with yield increases then the increase in yield potential of new varieties is likely to result in a greater lodging risk, unless the strength of the stem base and anchorage system is increased or crop height is reduced further by the use of breeding or use of chemical plant growth regulators [22].

In this study, 14 rice mega varieties (including 5 super high yield varieties) disseminated in China during the last 70 years were grown in the paddy field, and changes in lodging resistance and lodging-related morphological traits and their relationships with grain yield during genetic improvement were investigated. The objectives of this study were to: (1) determine the changes in stem lodging resistance and lodging-related morphological traits of mid-season rice during the last 70 years, (2) examine the relationships between the lodging-related traits of stem and grain yield, (3) identify the key morphological traits associated with lodging resistance.

## Materials and Methods

### Field experiment

Field experiments were conducted in farmers’ fields at Zhougan village (29°51_N, 115°53_E), Hubei Province, in the Middle Reaches of Yangtze River of China, during the middle rice growing season from May to October in 2013 and 2014 (The farmers’ fields at Zhougan village (29°51_N, 115°53_E), Hubei Province, are used as a experiment station for our college, which is permitted by local government. I confirm that our field studies did not involve any endangered or protected species). The soil of the experiment field had the texture of clay loam with pH 5.47, organic matter 29.10 g kg^−1^, total N 2.2 g kg^−1^, available P 12.14 mg kg^−1^ and available K 92.2 mg kg^−1^. Fourteen mega rice varieties that were released from 1936 to 2005 were used in this study, and they were planted in a large-scale area during the last 70 years in China. Among them, the seeds of Shenglixian (SLX), Aizizhan (AZZ), Guangchang’ai (GCA), Zhenzhu’ai (ZZA), Ezhong2 (EZ2), Guichao2 (GC2) were provided by China National Rice Research Institute in Zhejiang Province, China. Shanyou63 (SY63), Teqing (TQ) and IIyou725 (IIY725) were obtained from Daijin Agricultural Technology Center (Wuxue, Hubei, China). The seeds of Liangyoupeijiu (LYPJ), Yangliangyou6 (YLY6), Huanghuazhan (HHZ) and Yliangyou1(YLY1) were sourced from Hubei Provincial Seed Group Company. The information of these varieties was shown in Table 1.

**Table 1:** Information of rice varieties released in different ages since 1930s in China.

Variety	Abbreviation	Year of release	Type
Shenglixian	SLX	1936	inbred
Aizizhan	AZZ	1948	inbred
Guangchang’ai	GCA	1959	inbred
Zhenzhu’ai	ZZA	1962	inbred
Nanjing11	NJ11	1967	inbred
Ezhong2	EZ2	1969	inbred
Guichao2	GC2	1973	inbred
Shanyou63	SY63	1981	hybrid
Teqing	TQ	1984	inbred
IIyou725	ⅡY725	1995	superhybrid
Liangyoupeijiu	LYPJ	1999	superhybrid
Yangliangyou6	YLY6	2001	superhybrid
Huanghuazhan	HHZ	2005	inbred
Yliangyou1	YLY1	2005	superhybrid

The information was from the website of China Rice Data Center (http://www.ricedata.cn/variety/varis/600877.htm).

The experiment was arranged in a randomized complete block design with four replications. Totally there were 56 plots and the plot size was 30 m^-2^ (5 m×6 m). Pre-germinated seeds were sown in seedbed. Twenty-five-day old seedlings were transplanted on 9 June in 2013 and 6 June in 2014. The planting density was 25 hills m^-2^ at a hill spacing of 30 cm×13.3 cm with three seedlings per hill. Fertilizers (urea for N, single superphosphate for P and potassium chloride for K) were applied at the rates of 150 kg N ha^-1^, 40 kg P ha^-1^ and 100 kg K ha^-1^. N fertilize was split-applied at the ratio of 4:2:4 at basal (1 day before transplanting), tillering (7 days after transplanting), and panicle initiation stage. P and K were all applied at basal. The experimental field was kept flooding from transplanting until 7 days before maturity. Pests and weeds were controlled using chemicals to avoid yield losses.

### Measurements

**Fig 1:**
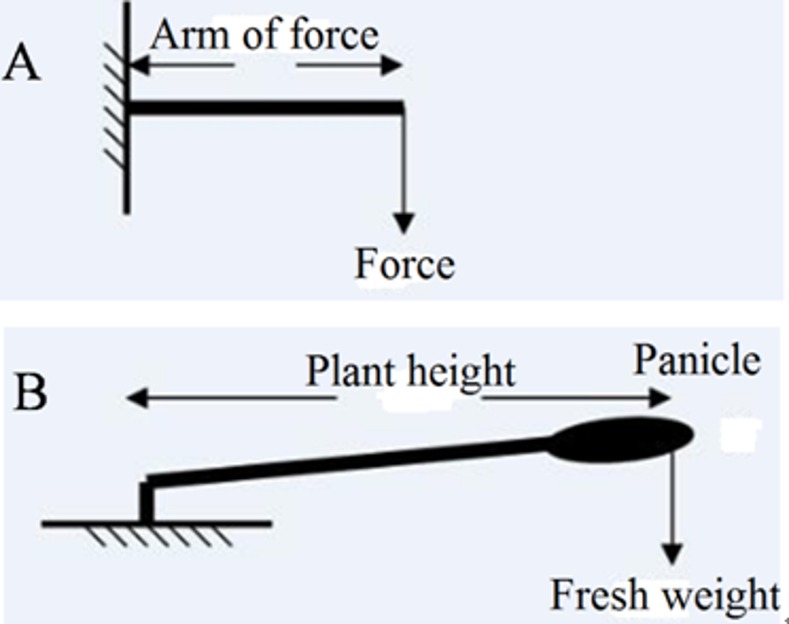
The schematic of bending moment (BM) was calculated using the following formula, BM = SL (stem length)×FW (fresh weight). Then, lodging index (bending moment/breaking resistance×100) was calculated for N_3_ internode according to Amano et al. (1993) [23]. The diameter of N_3_ near the lower node was measured after removing the leaf sheath. The dry weight of N_3_ with leaf sheath was measured after oven drying at 80°C to constant weight. Dry weight of N_3_ with leaf sheath per unit internode length (g cm^−1^) was also calculated [14, 24].

Lodging severity at maturity was scored visually on a scale of 0–9 where 0 was totally upright and 9 was totally lodged. Culm characters related to lodging were determined at 15 days after heading stage for each variety. Five representative hills were sampled from each plot and the 10 largest tillers (2 from each hill) were used to measure lodging-related traits. Stem length from the base of N_3_ internode (the third internode from top) to the tip of the panicle (SL), and the length of N_3_ internode (NL) were measured. The plant was cut at the lower node of N_3_ internode, and the breaking resistance of the middle point of N_3_ with leaf sheath was measured using a Prostrate Tester (DIK 7400, Japan). The distance between fulcra of the tester was set at 5 cm. The center of the internode, where breaking resistance was measured, was aligned horizontally with the middle point between the two fulcra. After measuring the breaking resistances of N_3_, fresh weights of the upper portion of the plant from the base of N_3_ internode including leaf and panicle (FW) were measured. Bending moment (BM) was calculated using the following formula, BM = SL×FW (In physics, the calculating formula for moment (M) is (Fig 1A): M = Force×Arm of force. Rice lodging can be simplified as the following model (Fig 1B), there is a hypothesis that fresh weight is all the weight on the top of the spikelets. Similarly, the calculating formula for bending moment (BM) is calculated as: BM = Plant height×Fresh weight).

### Statistical analysis

Analysis of variance (ANOVA) was performed using Statistix 9 (Analytical Software, FL, USA) and means of cultivars were compared based on the least significant difference (LSD) at the 0.05 probability level. Relationships among lodging-related traits and their relationships with year of release and gain yield were evaluated using correlation analyses (Statistix, 2009). The figure was generated by SigmaPlot 10.0 (SPSS Inc., Point Richmond, CA, USA).

## Results

**Fig 2:**
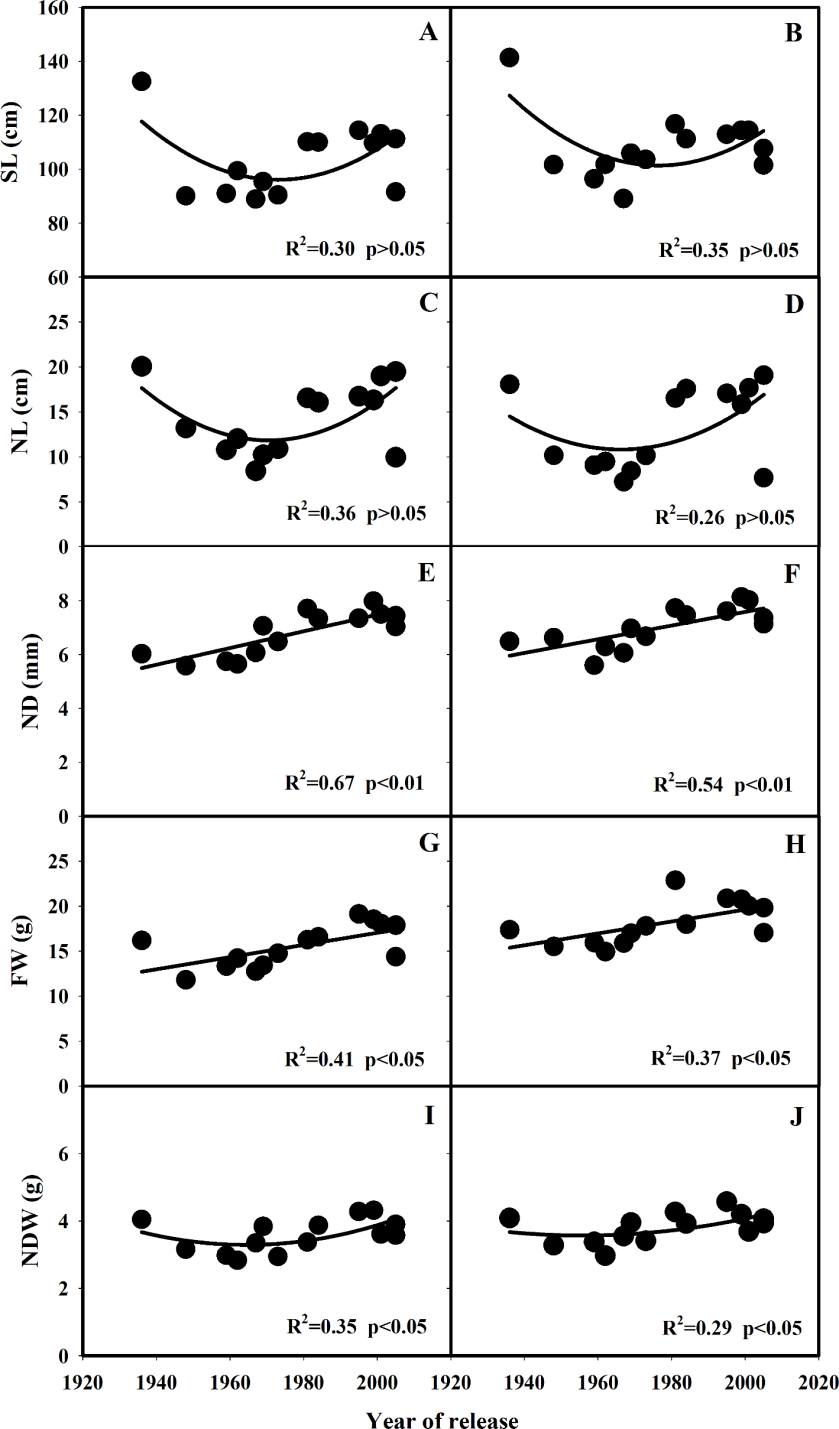
Changes in SL (A, B), NL (C, D), ND (EF, f), FW (G, H) and NDW (I, J) with the year of release of the varieties grown in 2013 and 2014. SL: stem length from the base of N_3_ internode to the tip of the panicle, NL: N_3_ internode length, ND: N_3_ internode diameter, FW: stem fresh weight, NDW: N_3_ internode dry weight.

Significant genotypic variation was observed in stem length (SL) in both years (Table 2). SL of SLX were 132.5 and 141.4 cm in 2013 and 2014, respectively, which were significantly higher than that of the other varieties. In general, there was a decreasing trend in SL for the varieties before 1980, but an increasing trend, although not statistically significant, for the varieties after 1980s (Fig 2A and 2B). Similar trend was observed for the N_3_ internode length (NL) in the process of genetic improvement (Fig 2C and 2D), except that NL of the varieties released after 1980s (except for HHZ) had similar values with SLX (Table 2). There was a significant increasing trend (*p*<0.05) in N_3_ internode diameter (ND) and stem fresh weight (FW) along with the release year of the varieties in both 2013 and 2014 (Table 2; Fig 2E, 2F, 2G and 2H). ND and FW of the varieties released after 1980s tended to be higher than that of the older varieties (Table 2). No significant differences (*p*>0.05) were observed in N_3_ internode dry weight (NDW) among these varieties in both 2013 and 2014 (Table 2; Fig 2I and 2J).

**Table 2:** Stem length (SL), N_3_ internode length (NL), N_3_ internode diameter (ND), Stem fresh weight (FW) and N_3_ internode dry weight (DW) of rice varieties grown in 2013 and 2014 released in different ages since 1930s in China.

Variety	SL (cm)		NL (cm)		ND (mm)		FW (g)		NDW (g)	
	2013	2014	2013	2014	2013	2014	2013	2014	2013	2014
SLX	132.5	141.4	20.1	18.1	6.1	6.5	16.2	17.4	4.0	4.1
AZZ	90.1	101.7	13.2	10.2	5.6	6.6	11.8	15.5	3.2	3.3
GCA	91.0	96.4	10.8	9.1	5.7	5.6	13.3	15.9	3.0	3.4
ZZA	99.4	101.9	12.0	9.5	5.7	6.3	14.2	14.9	2.8	3.0
NJ11	88.9	89.1	8.4	6.8	6.1	6.1	12.8	15.9	3.4	3.6
EZ2	95.4	106.0	10.2	8.4	7.1	7.0	12.6	17.0	3.8	4.0
GC2	90.5	103.6	10.4	7.9	6.5	6.7	14.8	17.8	2.9	3.4
SY63	110.2	116.8	16.5	17.3	7.7	7.8	16.3	22.7	3.4	4.3
TQ	110.1	111.3	16.1	18.1	7.4	7.5	16.6	18.0	3.9	3.9
ⅡY725	114.4	113.0	16.8	17.1	7.3	7.6	19.1	20.9	4.3	4.6
LYPJ	109.7	114.4	16.3	15.9	8.0	8.1	18.5	20.8	4.3	4.2
YLY6	113.1	114.4	19.0	17.7	7.5	8.0	18.0	20.0	3.6	3.7
HHZ	91.6	101.6	9.9	7.7	7.5	7.2	14.4	17.0	3.6	4.1
YLY1	111.3	107.7	19.5	19.1	7.1	7.4	17.9	19.8	3.9	3.9
LSD (0.05)	3.56	7.67	2.15	2.46	1.0	0.7	1.64	2.90	1.1	1.3

**Fig 3:**
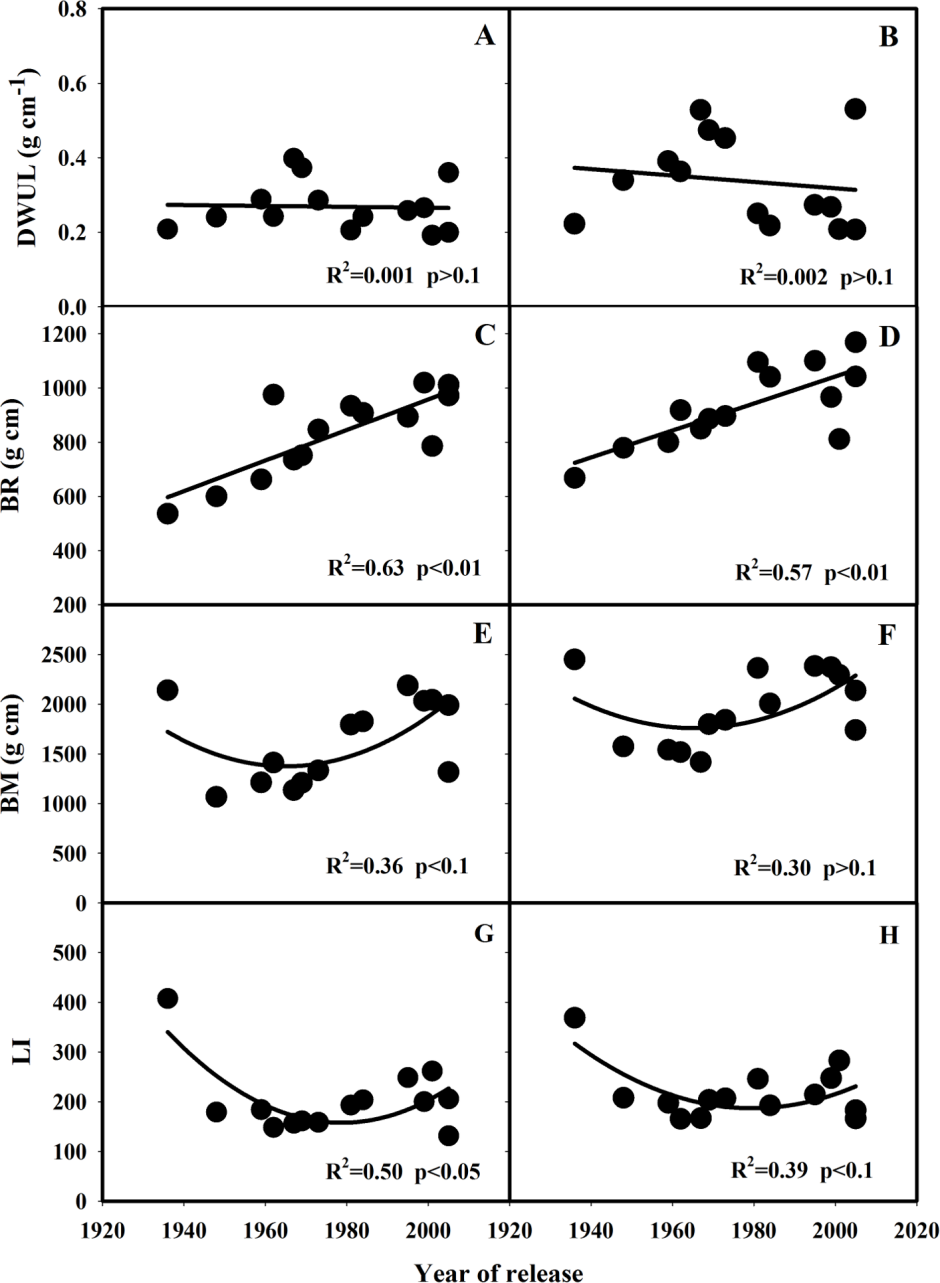
Changes in DWUL (A, B), BM (C, D), BR (E, F) and LI (G, H) with the year of release of the rice varieties grown in 2013 and 2014. DWUL: N_3_ internode dry weight per unit length, BM: bending moment, BR: breaking resistance, LI: lodging index.

Significant variation in N_3_ internode dry weight per unit length (DWUL) was observed among these varieties in both 2013 and 2014, however, the variation in DWUL was not significantly correlated with the year of release of these varieties (Table 3 and Fig 3A and 3B). DWUL of these varieties ranged from 0.18 to 0.38 g cm^-1^ in 2013 and 0.23 to 0.53 g cm^-1^ in 2014 (Table 3). Breaking resistance (BR) was significantly increased (*p*<0.05) along with the year of release in both 2013 and 2014 (Fig 3C and 3D). In both 2013 and 2014, SLX had the lowest BR of 536 and 668 g cm, respectively (Table 3). A decreasing trend for bending moment (BM) for varieties released from 1930s to 1970s were observed, and then BM was increased to similar value with SLX (Fig 3E and 3F). The values of BM ranged from 1067 to 2188 g cm in 2013 and 1418 to 2450 g cm in 2014 (Table 3). Significant variation in lodging index (LI) was observed among these varieties (*p*<0.05), and SLX had the highest values of 408 in 2013 and 369 in 2014 (Table 3). Variation in LI for the other varieties except for SLX was also statistically significant (*p*<0.05), but no relationship with the year of release of the varieties was observed (Fig 3G and 3H). Based on the visual score of lodging (VSL), SLX was severe lodging, and GC2, SY63 and YLY1 had occurred of lodging with medium extent in both years. For AZZ, GCA, ZZA, TQ and YLY6, lodging occurred in one of the two years (Table 3).

**Table 3:** Dry weight per unit length of N_3_ internode (DWUL), Bending Moment (BR), Breaking Resistance (BR), Lodging Index (LI) and Visual Score of Lodging (VSL) of rice varieties grown in 2013 and 2014 released in different ages since 1930s in China.

Variety	DWUL (g cm^-1^)		BM (g cm)		BR (g cm)		LI		VSL	
	2013	2014	2013	2014	2013	2014	2013	2014	2013	2014
SLX	0.20	0.23	2140	2450	536	668	408	369	8	8
AZZ	0.23	0.33	1067	1575	601	779	179	208	0	7
GCA	0.28	0.38	1213	1542	663	780	184	198	3	0
ZZA	0.25	0.35	1413	1519	975	918	148	166	0	4
NJ11	0.38	0.53	1135	1418	735	850	156	167	0	0
EZ2	0.35	0.50	1209	1799	752	886	161	204	0	0
GC2	0.28	0.45	1333	1842	847	897	159	207	3	7
SY63	0.23	0.25	1795	2664	934	1096	193	246	5	5
TQ	0.25	0.23	1827	2006	908	1041	204	192	3	0
ⅡY725	0.25	0.28	2188	2384	894	1100	249	214	0	0
LYPJ	0.28	0.28	2033	2374	1020	967	200	248	0	0
YLY6	0.18	0.23	2045	2296	786	812	261	283	0	3
HHZ	0.35	0.53	1317	1739	1012	1042	131	166	0	0
YLY1	0.20	0.23	1990	2137	972	1169	205	183	4	5
LSD (0.05)	0.10	0.15	207	378	125	163	44	39	-	-

Lodging score: 0–9, resistant to susceptible.

Correlation among the parameters related with lodging was shown in Table 4. LI was significantly correlated with SL, NL, FW and BM positively, but with DWUL and BR negatively in both 2013 and 2014 (Table 4). ND and FW both significantly correlated with BR in 2013 and 2014, while NDW and BM significantly correlated with BR only in 2014 (Table 4). Significantly positive correlation (*p*<0.05) was observed between BM and SL, NL, ND, FW and NDW in both 2013 and 2014, while negative correlation was observed between BM and DMUL in both 2013 and 2014 (Table 4).

**Table 4:** Correlation coefficient (r) among lodging-related traits of rice varieties grown in 2013 and 2014 in different ages since 1930s in China.

Parameters	SL	NL	ND	FW	NDW	DWUL	BM	BR
**2013**								
NL	0.85**							
ND	0.29*	0.29*						
FW	0.70**	0.68**	0.46**					
NDW	0.41**	0.29*	0.34*	0.33*				
DWUL	-0.43**	-0.66**	0.05 ^ns^	-0.33*	0.44*			
BM	0.89**	0.82**	0.41**	0.95**	0.38**	-0.41**		
BR	-0.04 ^ns^	0.01 ^ns^	0.44*	0.41**	0.09^ns^	0.11^ns^	0.22^ns^	
LI	0.84**	0.65**	0.02 ^ns^	0.45*	0.30**	-0.37**	0.67**	-0.51**
**2014**								
NL	0.64**							
ND	0.37**	0.52**						
FW	0.40**	0.58**	0.63**					
NDW	0.26*	0.26*	0.36**	0.50**				
DWUL	-0.46**	-0.79**	-0.26 *	-0.31*	0.26*			
BM	0.76**	0.71**	0.63**	0.90**	0.49**	-0.43**		
BR	-0.04 ^ns^	0.24 ^ns^	0.46 *	0.56**	0.38**	-0.01^ns^	0.39**	
LI	0.82**	0.51**	0.20 ^ns^	0.36**	0.14 ^ns^	-0.42**	0.64**	-0.44**

Levels of significance indicated: ns = not significant. NL: N_3_ internode length, ND: N_3_ internode diameter, FW: stem fresh weight, NDW: N_3_ internode dry weight, DWUL: N_3_ internode dry weight per unit length, BM: bending moment, BR: breaking resistance, LI: lodging index.

*significant atP≤0.05

**significant atP≤0.01. SL: stem length from the base of N_3_ internode to the tip of the panicle

**Fig 4:**
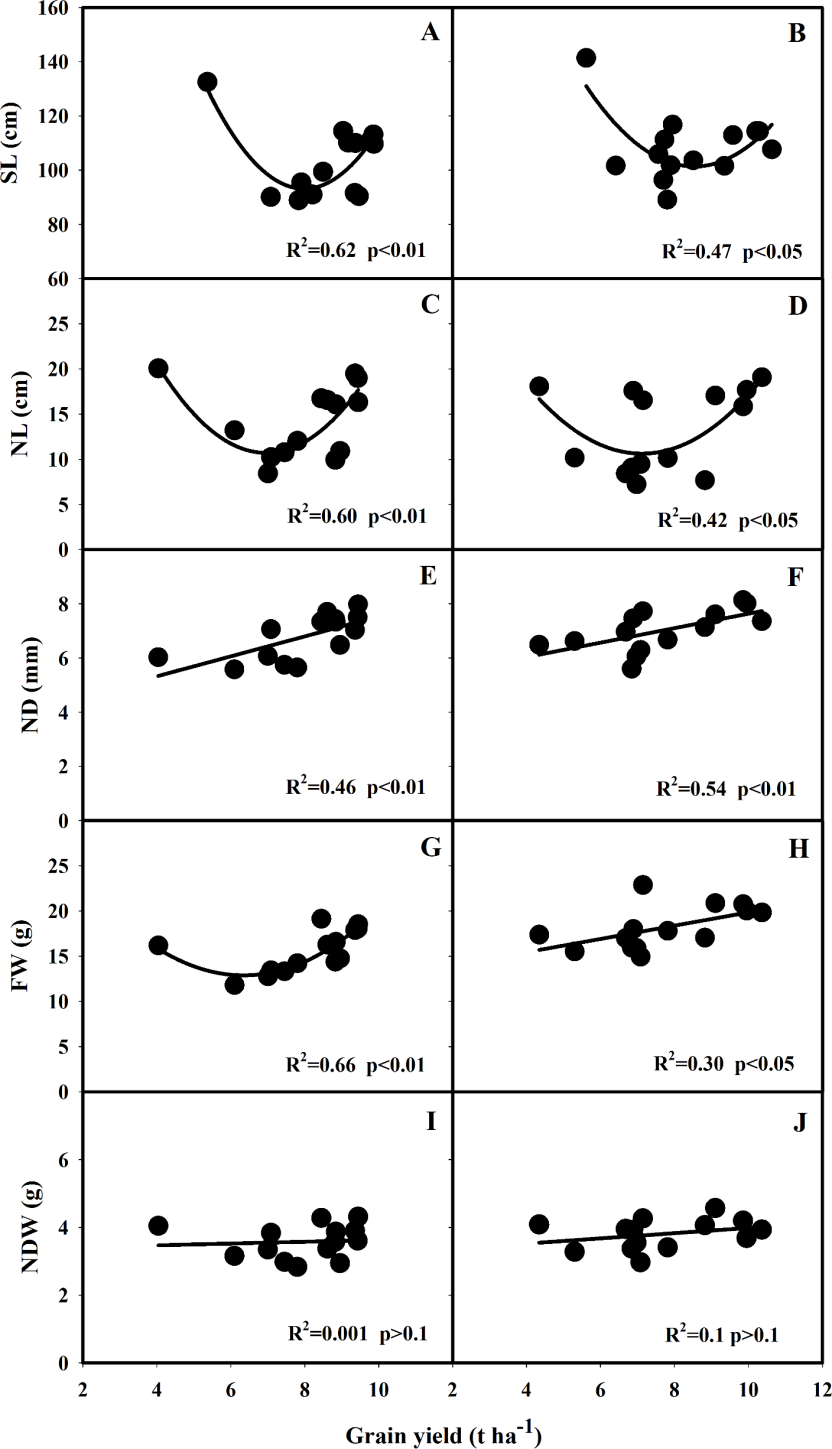
The correlation between grain yield and SL (A, B), NL (C, D), ND (E, F), FW (G, H) and NDW (I, J) of the rice varieties grown in 2013 and 2014. SL: stem length from the base of N_3_ internode to the tip of the panicle, NL: N_3_ internode length, ND: N_3_ internode diameter, FW: stem fresh weight, NDW: N_3_ internode dry weight.

**Fig 5:**
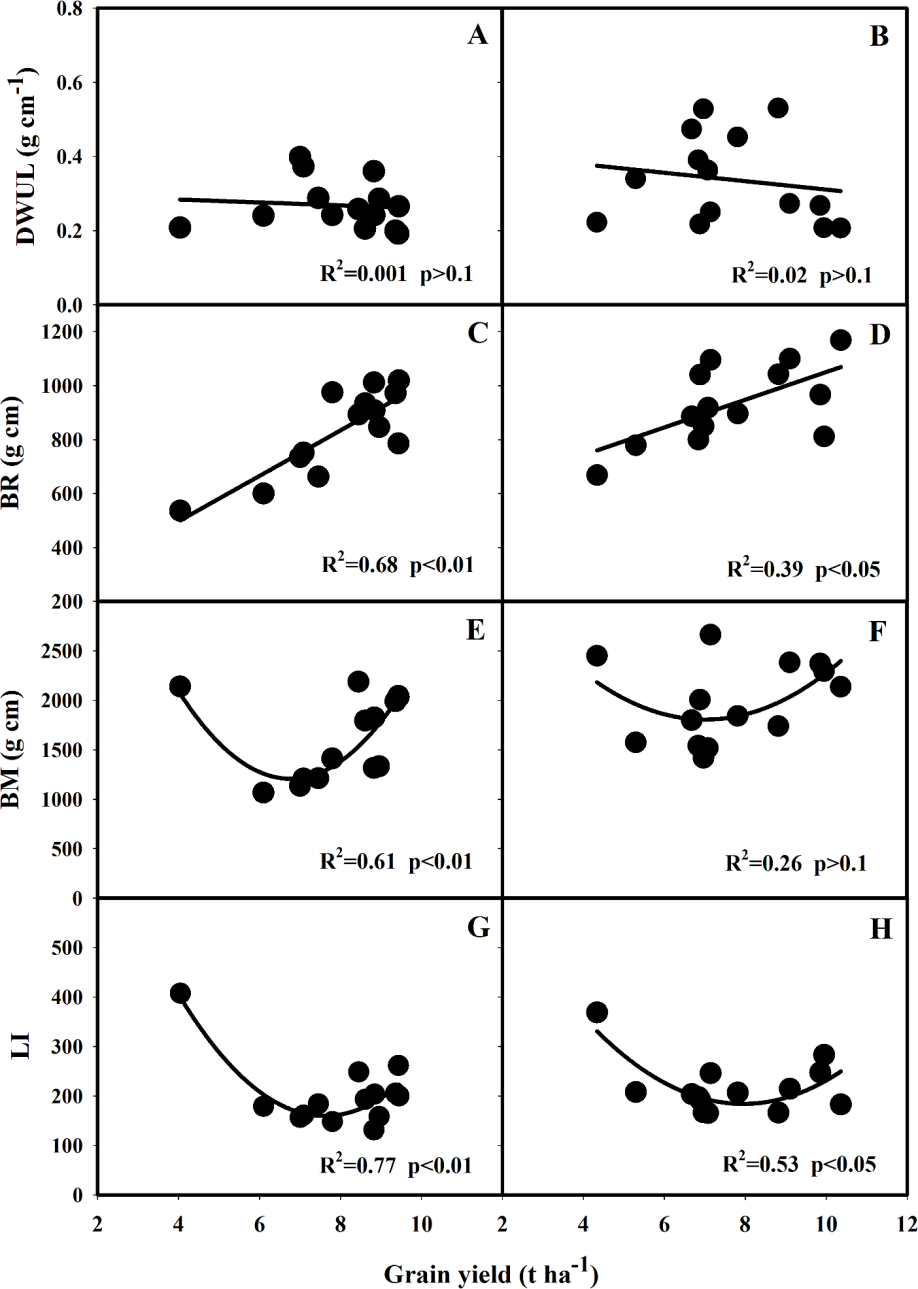
The correlation between grain yield and DWUL (A, B), BM (C, D), BR (E, F) and LI (G, H) of the rice varieties grown in 2013 and 2014. DWUL: N_3_ internode dry weight per unit length, BM: bending moment, BR: breaking resistance, LI: lodging index.

Lodging-related traits were correlated with grain yield in 2013 and 2014, which was shown in Figs 4 and 5. Significant quadratic relationships (*p*<0.05) between SL and grain yield, and between NL and grain yield were observed in both 2013 and 2014 (Fig 4A, 4B, 4C and 4D). Grain yield increased with the drastic reduction in SL after 1940s, and then with slightly increase in SL after 1980s (Fig 4A and 4B). Grain yield was significantly (*p*<0.05) correlated with ND and FW in both 2013 and 2014 (Fig 4E, 4F, 4G and 4H). No significant relationship was observed between NDW and grain yield (Fig 4I and 4J). There were significant differences in DWUL among these varieties, but no relationship between changes in DWUL and grain yield (Fig 5A and 5B). Significant linear correlation between BR and grain yield was found in 2013 and 2014 (Fig 5C and 5D), but no consistent relationship between BM and grain yield was observed in the two years (Fig 5E and 5F). Significant quadratic relationship between LI and grain yield was observed in both years, suggesting that increase in grain yield was associated with a decrease in lodging index (Fig 5G and 5H).

## Discussion

Grain yield was greatly improved due to the genetic improvement and the adoption of optimized crop management practices[25–26]. The national average rice grain yield has risen to about 6.4 t ha^-1^ in China in 2000’s, which has greatly contributed to food security in China[27–28]. New rice hybrids with high grain yield potential have been developed and are being grown on a large scale. According to our previous study, grain yield was increased from 4 ha^-1^ to 10 ha^-1^ with the genetic progress, and the increases in biomass production and sink size (spikelets per panicle) were responsible for the grain yield improvement[25, 29–30]. However, lodging is frequently occurred leading to reduction in grain yield [7], especially in high-yielding environments[14]. The reduction in grain yield due to lodging could be up to 50%[31]. Meanwhile, the occurrence of lodging resulted in deterioration of grain quality[20].

In order to reduce the occurrence of lodging in rice production, breeders have utilized the semi-dwarfing genes to produce shorter varieties[32]. This was reflected from the decrease in stem length and internode length when compared with a tall variety SLX (Fig 2A–2D). The adoption of semi-dwarfing rice varieties has significantly increased grain yield potential of rice, however, increasing evidences suggest that further increase in rice yield potential is restricted by plant height[21]. Therefore, breeders tended to select varieties with increased plant height after 1980s to further increase grain yield (Fig 4A and 4B)[17, 33–37].

LI of the short varieties was significantly reduced compared with that of SLX, while no significant (*p*>0.05) trend was observed in the changes of LI along with the year of release of the varieties except for SLX (Fig 3G and 3H). In the present study, determine coefficient of the correlation between LI and BM was larger than that between LI and BR, indicating that BM was more related with the changes in LI along with the year of release of these varieties (Table 4). Although BM of new varieties developed after 2000s was similar with that of SLX, LI of these new varieties was significantly lower than that of SLX due to the significantly higher BR (Fig 3C–3F). This is consistent with Islam et al. (2007) that plant height was not necessarily the most important factor determining LI[14]. Stem diameter, stem weight, leaf sheath wrapping, basal internode diameter and the cross-section area of the culm are the major traits affecting LI[38–39]. The N3 internode diameter and stem fresh weight were significantly increased along with the year of release (*p*<0.05), especially after 1980s (Fig 1E–1H). No significant change (*p*>0.05) was observed in N3 internode dry weight and dry weight per unit of length (Fig 2I and 2J and Fig 3A and 3B).

According to the Visual Score of Lodging (VSL) in both year's experiment (Table 3), the green revolution significantly reduced lodging risk in semi-dwarf rice variety. The tall variety of SLX had the maximum VSL in both years, which was attributed to their slender stature. The lodging score was much lower in semi-dwarf rice variety compared with SLX, however, semi-dwarf rice variety, such as AZZ, GCA and ZZA, was also medium lodged in 2013 or 2014 (Table 3). This was because the lower BR in AZZ (601 and 779 g cm) and GCA (663 and 780 g cm), and the lower NDW in ZZA (2.8 and 3.0 g). As for the modern rice variety, the lodging was due to the overweight in upper part of the plant. The enlarged sink size of modern rice variety with great yield potential resulted from larger panicles or heavy panicles[6, 25, 30], which exerted greater pressures to the lower internode. Although both ND and BR of modern rice varieties were enhanced significantly with year of release (Fig 2E and 2F and Fig 3C and 3D), the increased SL, NL, FW and BM may counteract their resistance contribution (Figs 2 and 3). So the occurrence of lodging is common in hybrid and super-hybrid rice production[14], especially under high-yielding environments[7]. The variation of the lodging-related traits in same variety in the 2 year's experiment like NDW, VSL was due to the big difference in weather condition in 2013 and 2014. The average daily maximum and minimum temperature during the growing season was 31.5 and 23.2°C in 2013, respectively, and 30.1 and 22.5°C in 2014, respectively. The mean daily solar radiation from May to October was 16.4 and 13.3 MJ m^−2^ day^−1^ in 2013 and 2014, respectively. The average daily maximum and minimum temperature during the growing season was 1.4 and 0.7°C higher in 2013 than in 2014, respectively. The mean daily solar radiation from May to October in 2013 was 23.3% higher than that in 2014. The weather condition was introduced in our previous study [30]. In addition, there was a big storm during grain filling stage in 2014. So, some varieties showed poor repeatability in our study, such as AZZ showed 7 VSL in 2014 but 0 in 2013. And same situation was showed in other study [14].

Correlation analysis showed that SL and NL significant positive correlated with LI (Table 4). This is inconsistent with the results of Islam et al. (2007) that there was no correlation between culm height and lodging index of lower internodes[14]. Plant height was not necessarily the most important factor in determining lodging resistance[40], but long culm length and large leaf area index of hybrid rice may cause an increase in bending moment[41], resulting in high lodging index[14]. In addition, DWUL was negative correlated with LI significantly (Table 4) despite NDW and DWUL was not enhanced with year of release. Studies have shown that both dry weight of internode per unit length and internode diameter contributed to the high breaking resistance of lower internodes[14]. What is more, internode dry weight has been reported to be negatively correlated with the lodging score in the field and positively correlated with the breaking strength of wheat straw[39]. These results suggest that further increase in ND, NDW and DWUL of lower internode in modern super rice variety by genetic could effectively enhance lodging resistance and bring down LI. Therefore, it is a feasible pathway to improve the lodging resistance of super rice by selecting genotypes with high BR, high ND, high NDW, high DWUL, and low LI at lower internodes.

## Conclusion

Significant differences in the lodging-related traits were observed along with the release year of the varieties. Lodging index was significantly reduced with the adoption of semi-dwarf varieties. There was no significant relationship between LI and the release year for the semi-dwarf varieties, although significant differences in LI was observed among these varieties. Significantly higher breaking resistance contributed to maintain a lower LI for the newer varieties with higher plant height and similar breaking moment with SLX. Internode diameter and stem fresh weight were significantly increased along with the release year of the varieties, which resulted in the significantly increasing BR.
